# Computational Methodologies in the Exploration of Marine Natural Product Leads

**DOI:** 10.3390/md16070236

**Published:** 2018-07-13

**Authors:** Florbela Pereira, Joao Aires-de-Sousa

**Affiliations:** LAQV and REQUIMTE, Departamento de Química, Faculdade de Ciências e Tecnologia, Universidade Nova de Lisboa, 2829-516 Caparica, Portugal

**Keywords:** Computer-Aided Drug Design (CADD), drug discovery, chemoinformatics, bioinformatics, machine learning (ML), marine natural products (MNPs)

## Abstract

Computational methodologies are assisting the exploration of marine natural products (MNPs) to make the discovery of new leads more efficient, to repurpose known MNPs, to target new metabolites on the basis of genome analysis, to reveal mechanisms of action, and to optimize leads. In silico efforts in drug discovery of NPs have mainly focused on two tasks: dereplication and prediction of bioactivities. The exploration of new chemical spaces and the application of predicted spectral data must be included in new approaches to select species, extracts, and growth conditions with maximum probabilities of medicinal chemistry novelty. In this review, the most relevant current computational dereplication methodologies are highlighted. Structure-based (SB) and ligand-based (LB) chemoinformatics approaches have become essential tools for the virtual screening of NPs either in small datasets of isolated compounds or in large-scale databases. The most common LB techniques include Quantitative Structure–Activity Relationships (QSAR), estimation of drug likeness, prediction of adsorption, distribution, metabolism, excretion, and toxicity (ADMET) properties, similarity searching, and pharmacophore identification. Analogously, molecular dynamics, docking and binding cavity analysis have been used in SB approaches. Their significance and achievements are the main focus of this review.

## 1. Introduction

**Figure 1:**
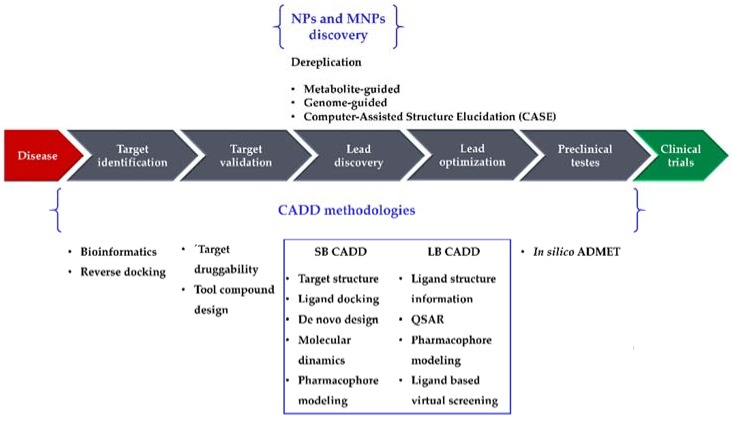
The drug discovery pipeline, computer-aided drug design (CADD), and natural product (NP)/marine natural product (MNP) discovery methodologies. SB, structure-based; LB, ligand-based; ADMET, adsorption, distribution, metabolism, excretion, and toxicity; QSAR, Quantitative Structure–Activity Relationship.

Drug research and development (R&D) is comprehensive, complex, expensive, time-consuming, and full of risk. A 2016 study [1] reported a clinical success rate, i.e., the likelihood that a drug that enters clinical testing will eventually be approved, of approximately 12%. The development of a drug from concept to market currently takes 13–15 years and requires United States $2–3 billion on average [2]. Although such costs are going up, the number of drugs approved every year per billion dollars spent on R&D has remained flat or decreased for most of the past decade [3]. Several new methodologies have been developed and applied in drug R&D to shorten the research cycle and to reduce the costs. Computational methodologies have been instrumental at various stages of drug discovery [4,5] and continue to be indispensable in the incessant demand for life-saving drugs. Computer-Aided Drug Design (CADD) methods have emerged as a powerful tool in the development of therapeutically important small molecules for over three decades [6,7,8], enabling higher hit rates than experimental high-throughput screening (HTS) approaches alone [6]. For example, Mueller et al. [6] built a computational model using results from a previous HTS of metabotropic glutamate receptor 5 (mGlu5) activity [9], which was able to identify new lead-like mGlu5 modulators in a virtual screening experiment with a hit rate of 3.6% [6]: an enrichment factor of approximately 16 compared with the original experimental HTS data (0.22%) [9]. Nowadays, CADD methodologies have been extended from their more conventional application of lead discovery and optimization toward new directions, e.g., target identification and validation and preclinical tests (prediction of adsorption, distribution, metabolism, excretion, and toxicity (ADMET) properties). They are generally classified in two categories, structure-based (SB) and ligand-based (LB), both of which have been used with marine natural products (MNPs). In this review, we highlight recent advances of CADD methodologies applied to NPs (particularly focusing on MNPs), as well as the importance of informatics in the analysis of marine extracts from an early stage of screening, to recognize and filter out known compounds (dereplication) [10,11]. Figure 1 illustrates the role of computational methodologies in a typical drug discovery pipeline and the importance of NPs and MNPs in this context.

**Figure 2:**
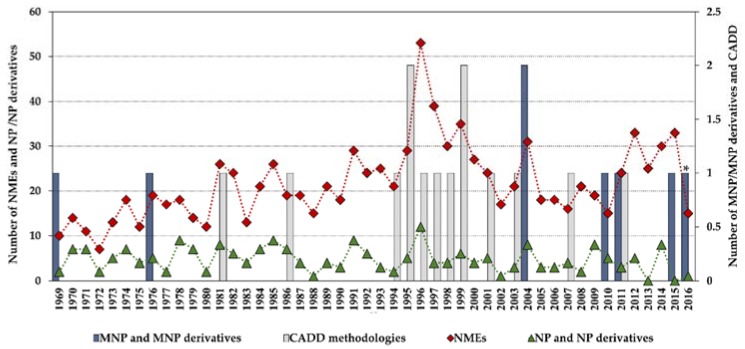
Novel Food and Drug Administration (FDA) approvals during 1969–2016, where new molecular entities (NMEs) are all approvals except biologics license applications; NP and derivatives are all non-mammalian NPs except MNPs; CADD methodologies are approvals that were developed using CADD; * an MNP that is an European Medicines Agency (EMEA)-approved drug. Data are from Drugs@FDA and the literature [7,12,13,14,15,16,17].

Statistics concerning novel drug approvals by the Food and Drug Administration (FDA) during 1969–2016 show a current downward trend since the high-point in 1996 (53 new molecular entities (NMEs)/year), and the minimum (after 1996) of 15 NMEs/year in 2010 and 2016. Figure 2 compares the global number of novel FDA approvals with the number of approvals of NP and derivatives between 1969 and 2016 and highlights the contribution of MNPs and CADD methodologies.

**Figure 3:**
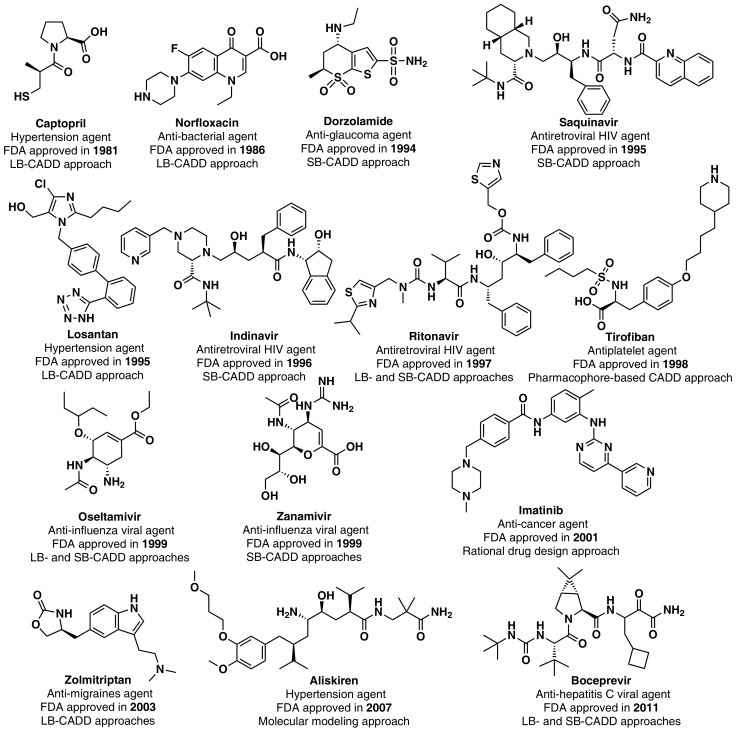
CADD-driven drugs and their chemical structures and clinical indication.

Although the high-point in the mid to late 1990s appears to be mainly due to regulatory factors [3] (i.e., clearing of a backlog at the FDA following the implementation of the 1992 Prescription Drug User Fee Act, and political lobbying for human immunodeficiency virus (HIV) drugs, which lowered the normal regulatory hurdles), major advances in many of the scientific and technological inputs into R&D had been accomplished during the 1980s and 1990s. For example, combinatorial chemistry increased the capability to produce drug-like molecules by approximately 800 fold, increasing the size of the known chemical space [3,18,19]; faster DNA sequencing allowed the identification of new drug targets [20]; advancements in the elucidation of three-dimensional protein structures via X-ray crystallography facilitated the identification of lead compounds through structure-guided strategies[21]; the advent of HTS led to an explosion in the rate of data generation [22]; and computational drug design and screening were implemented [7]. Interestingly, the high-point for NP and derivatives was also in 1996 (with 12 approved drugs) and the 1990s decade was also the most successful for CADD-driven drugs, with eight approved drugs (Figure 3).

**Figure 4:**
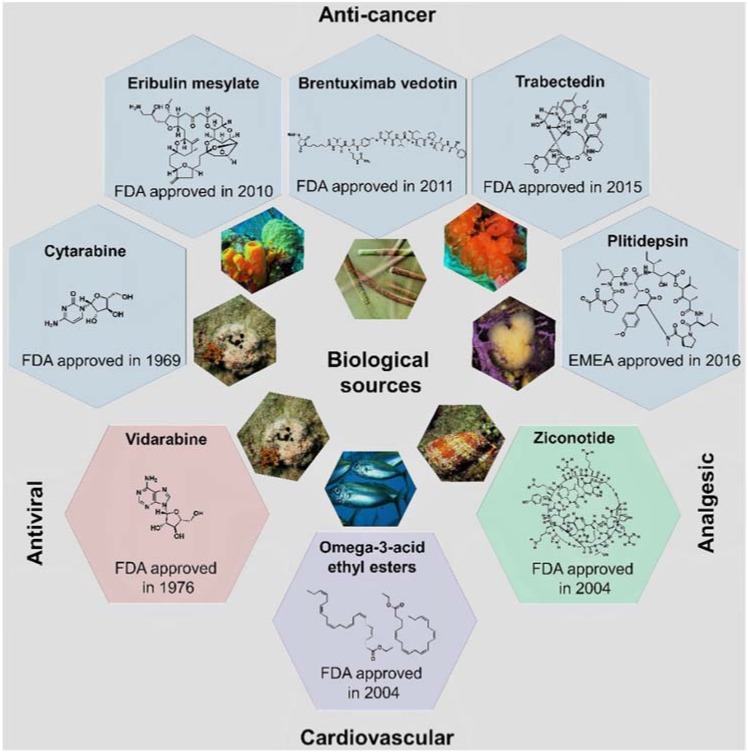
The eight approved MNP and derivative drugs and their biological sources, chemical structures, and clinical usage.

More than half of the total approvals of MNP and derivatives occurred in the 21st century (six out of eight approved drugs, Figure 4). The declining number of NMEs in development pipelines together with the higher success rate of marine compounds (1 in 3500 MNPs [13] against the industry average of 1 in 5000–10,000 compounds [23]) have led to the rekindling of interest in NP-like scaffolds [23,24]. More than 28,000 MNPs have been reported to date from a variety of marine sources (http://pubs.rsc.org/marinlit); in 2016, the literature reported 1277 new compounds [25] isolated from marine microorganisms and phytoplankton, green, brown, and red algae, sponges, cnidarians, bryozoans, molluscs, tunicates, echinoderms, mangroves, and other intertidal plants and microorganisms. However, only eight MNPs have to date been approved as drugs (Figure 2 and Figure 4), while 12 marine-derived metabolites are currently in different phases of clinical trials [25,26,27,28,29]. New approaches are needed to overcome the perceived disadvantages of NPs as compared with synthetic drugs, such as the difficulty in access and supply, the complexity of NP chemistry, and the inherent slowness of working with NPs [28,30]. Existing NP databases must be improved by filling missing activity records [31]. It shall be mentioned that the known biological activity space of MNPs has been biased due to funding sources, e.g., five out of the eight approved MNPs drugs are anti-cancer drugs (Figure 4). The emphasis on cancer is mainly due to the fact that the major funding agency in the U.S. for MNP and derivatives was for many years the National Institutes of Health (NIH)/National Cancer Institute (NCI), and this happened similarly in other countries [13].

The vast majority of currently used antibiotics have been isolated from terrestrial microbes, accounting for more than 75% of all antibiotics discovered [32,33], but antimicrobial compounds from marine sources have not yet been developed into clinical testing phases [13,28]. Recently, the marine environment has been proposed as an untapped source of new bioactive molecules, and marine bacteria and fungi seem to be the most important sources for antibacterial discovery [28,34,35,36]. Computational methodologies are crucial in the systematic exploration of the biological activity of MNPs to improve the rate of drug discovery from marine sources. Their significance, achievements, and challenges are addressed in this review.

## 2. Databases

Specific databases of NPs and MNPs are available with physical, chemical, and biological properties. Furthermore, databases of larger scope also include compounds from marine sources, as well as similar compounds from other sources, and are useful resources for the development of MNP leads. The exploration of databases has become a well-established essential component of chemistry and biological research. Some of these databases are just collections of chemical structures, e.g., catalogues of commercially available samples for screening, while others provide additional data, such as measured bioactivities and protein targets as well as targeted diseases. Only a fraction of large general databases is directly related to NPs, but some exist that can assist in NP-based drug discovery and dereplication. To be useful for dereplication purposes, databases must cover extensively the chemical and biological space of the known NPs and must be searchable by several features, such as structure and substructure identity/similarity, spectroscopic identity/similarity, UV absorption maxima, accurate mass, physical properties, taxonomic identification of the producing macro- or micro-organism, biological activity, and biological targets. For CADD procedures, databases must provide compounds with their molecular structures in chemical file formats, bioactivity data (e.g., cell-based assays), and biomolecular targets. They contain advantageously medicinal chemistry data, NP data, approved drugs and failed drug candidates with data generated in the preclinical and clinical phases of drug discovery [37,38,39]. The most relevant databases for NPs as well as their searchable attributes are listed in Table 1 (the ReSpect and NaprAlert databases have not been updated since 2012 and 2016, respectively).

Substructure searching is available for all databases reported in Table 1 with the exception of the NaprAlert and NPCARE databases. CAS/SciFinder, available at Scientific and Technical Network (http://www.cas.org/products/scifinder) is a commercial database comprising one of the largest online repository of NPs structures, although it has several search limitations to be applied in dereplication procedures (e.g., it does not allow one to search by spectral data or accurate mass). Other commercially available databases are: REAXYS, licensed by Elsevier B.V. (https://new.reaxys.com), which provides access to experimentally measured data (physical, chemical, and pharmacological data); ACD/NMR DB from ACD/Labs (http://www.acdlabs.com/products/dbs/nmr_db), which consists of experimental NMR spectra, currently including 210,000 ^1^H, >200,000 ^13^C, 16,780 ^19^F, 9200 ^15^N, and >27,000 ^31^P NMR spectra; NaprAlert (http://www.napralert.org); and the Chapman & Hall/CRC Dictionary of NPs (http://dnp.chemnetbase.com).

More specific databases particularly focusing on MNPs are the Chapman & Hall/CRC Dictionary of MNPs (http://dmnp.chemnetbase.com), MarinLit (http://pubs.rsc.org/marinlit/), and AntiBase (http://application.wiley-vch.de/stmdata/antibase.php). AntiBase covers terrestrial and marine microbial NPs and includes predicted ^13^C NMR spectra for compounds with no available experimental spectra.

The remaining databases listed in Table 1 are freely available. The StreptomeDB (http://www.pharmaceutical-bioinformatics.org/streptomedb/) is a versatile platform for the gathering of information concerning the genus *Streptomyces*, an actinobacteria that has stirred huge interest as a source of bioactive compounds over the last few decades; all molecular structures can be downloaded with metadata in the MDL SD file format [40] NPCARE (http://silver.sejong.ac.kr/npcare) is an online database of NPs and fractional extracts for anticancer activities, which were validated with 1107 cell lines for 34 cancer types [41]. Each record is annotated with the cancer type, the genus, and species names of the biological resource, the cell line used for demonstrating the anticancer activity, the PubChem ID, and information about the target gene or protein.

ChemSpider (http://www.chemspider.com) is a curated chemical database, which was made available from the Royal Society of Chemistry (RSC) and contains data for compounds gathered from over 500 different sources [42]. PubChem (http://pubchem.ncbi.nlm.nih.gov) is probably the largest freely available collection of chemical information and one of the largest repositories of NPs; it is organized as three interlinked databases (Substance, Compound, and BioAssay) [38] and includes more than 234 million depositor-provided chemical substance descriptions, 93 million unique chemical structures, and 1.2 million biological assay descriptions, covering about 10,300 and 22,000 unique protein target and gene target sequences, respectively. ChEMBL (http://www.ebi.ac.uk/chembl) is a large-scale curated bioactivity database with information on molecule–target interactions retrieved from the published literature; it has been expanded both in terms of data content (e.g., a neglected tropical disease archive including datasets from GlaxoSmithKline, Novartis, St. Jude Children´s Research Hospital , FDA-approved drugs, and drug candidates in clinical development) and annotation (e.g., properties and efficacy targets for FDA-approved drugs and drug candidates in clinical development) [37]. The ZINC (http://zinc15.docking.org/), LOPAC, and Prestwick databases comprise commercially available molecules, thus linking available collections of samples for experimental screening to known targets. LOPAC, available from Sigma-Aldrich, is a chemogenomic library that contains pharmacologically relevant small molecule agents and a complete list of compounds and their annotated targets (more than 450 targets); more than 50% of the compounds target G-protein-coupled receptor (GPCR), similarly to approved drugs, making it particularly well-suited to screen for GPCR-related phenotypic effects [43]. The Prestwick chemical library (http://www.prestwickchemical.com/prestwick-chemical-library.html) is also a chemogenomic library with mostly approved drugs that were selected for target diversity (more than 100 targets) and known safety and bioavailability [43]. ZINC is a free database designed to bring together biology and chemoinformatics; it is simultaneously easy to use by non-specialists and fully programmable for chemoinformaticians and computational biologists. The ZINC 15 version [44] was expanded from an exclusively molecule-centric database (mainly used for virtual screening, ligand discovery, pharmacophore screening, benchmarking, and force field development) to one that connects molecules to biological targets, processes, and other bioactive small molecules; the biological annotations, such as the identification of molecules as metabolites, drugs, and NPs and the identification of molecules as ligands for particular proteins and processes, were derived from other databases and libraries, e.g., HMDB [45], ChEMBL [37], and DrugBank [46]. Moreover, several NPs databases have also been incorporated in the ZINC database, namely: AfroDb [47], a database of NPs from African sources; HIM (Herbal Ingredients In-Vivo Metabolism database) [48]; NPACT (naturally occurring plant-based anti-cancer compound-activity-target database) [49]; NuBBE [50], a NPs database from the biodiversity of Brazil; and TCM database@Taiwan [51] with traditional Chinese medicine compounds. The use of databases in dereplication and CADD procedures is further discussed in Section 3.1.1 (Secondary-metabolite-guided identification) and Section 4.1 (Ligand-based CADD), respectively.

## 3. Dereplication

Dereplication involves the comparison of experimental data from new extracts with those of known NPs, and therefore computational methodologies associated with databases are essential to increase the chance of isolating new molecules efficiently. For reviews of the NP dereplication literature in general, the reader is referred to Gaudêncio and Pereira [10], Pérez-Victoria et al. [11], and Zhang et al. [52]. Mohamed et al. [53] reviewed computational resources for NPs dereplication, and Hufsky et al. [54] is suggested for a review of informatics methods for NP discovery. Here, we highlight the most relevant recent advances in computational dereplication methodologies employing computational mass spectrometry or NMR spectroscopy (metabolite-guided and genome-guided approaches) and computer-assisted structure elucidation (CASE), in particular those concerning MNPs or likely to be applied to MNPs. Genome mining is a strategy to aim at the isolation of novel NPs [55] as the identification of genes encoding for the biosynthesis of secondary metabolites can guide the exploration of extracts to identify anticipated new molecules.

### 3.1. Computer-Assisted Identification of Compounds

#### 3.1.1. Secondary Metabolite-Guided

Different analytical techniques, such as liquid chromatography-mass spectrometry (LC-MS) [56,57], liquid chromatography-high resolution mass spectrometry (LC-HRMS) [58,59,60], liquid chromatography time-of-flight MS (LC-TOF-MS) [61], high-resolution electrospray ionisation mass spectrometry (HRESI-MS) [62], and NMR spectroscopy [59,60,62], have been applied for fast dereplication followed by multivariate data analysis to minimize redundancy in the isolation steps.

**Figure 5:**
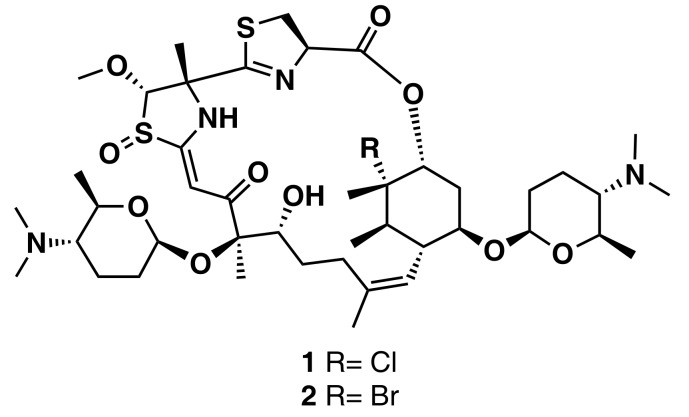
Chemical structures of the antifungal agents forazoline A (**1**) and B (**2**) isolated from an *Actinomadura* sp.

Chanana et al. [56] developed an LC-MS-based principal component analysis (PCA) workflow, which comprises a new script written in R (PoPCAR, Planes of Principal Component Analysis in R), to distinguish unique versus common metabolites in ~50 marine actinomycete strains. PoPCAR allows researchers to identify masses or molecules unique to each strain by locating those in a bucket table with a peak list, which can be generated using commercial software, such as Bruker ProfileAnalysis or open source tools, e.g., MZmine [63] or XCMS [64]. The AntiBase database was also integrated into this workflow. With this strategy, the authors were able to pinpoint the skeleton of forazoline, one of three classes of novel compounds previously identified from an *Actinomadura* sp. (Figure 5).

A similar approach was reported using PCA, hierarchical clustering (HCA), and orthogonal partial least square-discriminant analysis (OPLS-DA) to evaluate the high resolution Fourier transform mass spectrometry (HRFTMS) and NMR data of marine sponge-associated bacterium Actinokineospora sp. crude extracts, which were cultivated from the Red Sea sponge Spheciospongia vagabunda [60]. The differential analysis of sample populations was accomplished using the MZmine software; the MS and NMR records from the databases AntiBase and MarinLit were used to identify the known secondary metabolites. With this dereplication workflow, two new antiparasitic O-glycosylated angucyclines, actinosporins A and B, were identified.

Roullier et al. [65] highlighted the potential of marine-derived fungi for new bioactive metabolites and their under-investigated halogenated metabolome and focused on the detection of new halogenated compounds among a collection of marine-derived fungal strains. A new software tool, MeHaloCoA, was developed under R to automate the identification of halogenated compounds in HPLC-MS profiles and was demonstrated with the identification and isolation of two new MNPs from a Penicillium canescens strain, chlorogriseofulvine and griseophenone I, which exhibited antiproliferative activities.

The Global Natural Products Social Molecular Networking (GNPS; http://gnps.ucsd.edu) is an open-access knowledge base for community-wide organization and sharing of raw, processed, or identified tandem mass (MS/MS) spectrometry data. It provides access to spectral libraries, dereplication tools, and visualization of molecular networks based on spectral correlation [66]. Examples of a GNPS application with MNPs include the analysis of 146 marine Salinispora and Streptomyces strains [67] and the chemical profiling of the Alphaproteobacterium strain MOLA1416 associated with the marine lichen Lichina pygmaea [68].

Although the application of hyphenated analytical and statistical methods in metabolomics facilitates the discovery of potentially novel secondary metabolites from plant, animal, and microbial origin, there are still several challenges that have to be addressed in order to achieve a real leap forward in drug discovery from natural sources. For example, comprehensive MS and NMR databases are not available for small molecules; thus, compound deconvolution and identification often require extensive searching of individual databases. Most databases do not contain MS fragmentation spectra and two-dimensional (2D) NMR spectra, which are crucial for small molecule structure elucidation and unambiguous dereplication. Moreover, the NP drug discovery process can only be exponentially improved, in our opinion, with the inclusion of predicted spectral data using computational methods. In order to amplify the spectral data space, predicted spectra can be generated for known chemical spaces and for unknown chemical spaces exponentially amplified by automatic molecular structure generators [69].

The following examples illustrate these points. The PubChem database currently contains about 90 million compounds, while the two largest (commercial) MS spectral libraries, from the National Institute of Standards and Technology (version 17) and Wiley Registry (11th edition), enclose MS data for 267,000 and 741,000 compounds, respectively; the largest (commercial) NMR spectral database, ACD/NMR DB, comprises NMR data for ~322,000 compounds. Kerber et al. [69] reported that among more than 109 million possible molecular structures with the formula C_8_H_6_N_2_O (mass 146 Da), only 1911 hits matched in PubChem database.

Several strategies have been devised to explore this huge searchable chemical space. For example, Jeffryes et al. [70] used the Biochemical Network Integrated Computational Explorer (BNICE) and expert-curated reaction rules based on the Enzyme Commission classification system to compute Metabolic in silico Network Expansions (MINEs). This is an extension of databases with known metabolites to include molecules that have not yet been observed, but are likely to occur based on known metabolites and biochemical reactions. These databases are freely available from http://minedatabase.mcs.anl.gov. Recently, Lai et al. [71] reported the integration of a metabolome database, BinBase (a large GC-MS-based untargeted metabolomics database covering various species, organs, and matrices), with the mass spectrometry chemoinformatics tools BinVestigate (http://binvestigate.fiehnlab.ucdavis.edu), MS-DIAL 2.0, and MS-FINDER 2.0 (http://prime.psc.riken.jp). The goal is to annotate unknown metabolites modified by enzymatic transformations that gain physiological functions in a given biological system (epimetabolites) [71]. This methodology revealed that N-methyl-uridine monophosphate was highly upregulated in cancer cells and cancer tissues compared with its levels in any other cell type or tissue [71]. Another example is LipidBlast (http://fiehnlab.ucdavis.edu/projects/LipidBlast), a simulated mass spectral library for 119,200 compounds automatically generated from typical structural motifs of lipids [72].

The combination of molecular structure generators and spectra prediction methods for augmented spectral data spaces has been very successful in proteomics for many years as the prediction of peptide fragmentation patterns is easier. Hufsky and Böcker [73] reviewed the literature and identified four main approaches to mine a database of metabolite structures beyond a straightforward comparison of experimental spectra: (1) rule-based fragmentation spectrum prediction; (2) combinatorial fragmentation; (3) competitive fragmentation modelling; and (4) molecular fingerprint prediction. Rules for fragmentation prediction can be automatically learned from experimental data using machine learning (ML) techniques [74,75]. Kangas et al. [74] reported an algorithm, the so-called “in silico identification software (ISIS)”, which generates in silico spectra of lipids for the purpose of structural identification. This method uses artificial neural networks (ANN) to find accurate bond cleavage rates in a mass spectrometer employing collision-induced dissociation tandem mass spectrometry. Searching a database of 18,399 calculated spectra against the experimental spectra of 45 test lipids yielded the correct structure at the top position in 40 cases and at the second position in 5 cases.

In contrast to rule-based fragmentation, combinatorial fragmentation does not aim at predicting a mass spectrum but rather at explaining the peaks in the experimental fragmentation spectrum of a metabolite by matching against possible fragments enumerated with systematic bond dissociation, i.e., mapping fragmentation spectra to molecular structures. MetFrag [76] and MetFusion [77] are the most used tools for combinatorial fragmentation. MetFusion combines MetFrag results with a spectral library search in the MassBank database. More recently, the Metabolite Identification via Database Searching (MIDAS) algorithm was reported [78]. Similarly to MetFrag, MIDAS exhaustively enumerates possible fragments, but then calculates the plausibility of the fragments based on their fragmentation pathways, instead of bond dissociation energies, to evaluate a metabolite-spectrum match (MSM); the MSM score is calculated to reflect how well the metabolite explains the spectrum. MIDAS was designed to search high-resolution tandem mass spectra against a large metabolite database in an automated and high-throughput manner. It was tested with four standard ESI-MS/MS data sets from MassBank and revealed high accuracy in the identification of metabolites against the MetaCyc database, even outperforming MetFrag [78]. It was also demonstrated using a real-world LC-ESI-MS/MS measurement of a metabolome from Synechococcus sp. PCC 7002, a marine cyanobacterium: many metabolites previously found using spectral library searching, chemical formula matching, and manual interpretation were identified, but MIDAS additionally identified many other metabolites missed in the previous study. In a further development, Ridder et al. reported a substructure-based annotation of high-resolution multistage MS^n^ spectral trees (MAGMa), which uses the hierarchical information available from this technique to explain the fragment peaks observed at consecutive levels of the MS^n^ spectral tree [79,80]. The MAGMa+ software is available that combines MIDAS and MAGMa and uses metabolite-dependent optimized parameters obtained with ML techniques [81].

The competitive fragmentation modelling (CFM) approach [75] predicts mass spectra using a probabilistic generative model for the MS/MS fragmentation process and an ML approach for learning model parameters from experimental data. The fragmentation process is modelled as a stochastic homogeneous Markov process. This model estimates the likelihood of any given fragmentation event and predicts those peaks that are most likely to be observed, thus improving precision. It was shown that CFM can be used to predict the MS/MS spectrum from a chemical structure and to rank possible structures for an observed spectrum.

The FingerID method of Heinonen et al. [82] uses an ML approach to predict structural properties (fingerprints) of unknown molecules from their MS spectra rather than predicting fragmentation MS spectra from chemical structures. Then, the predicted fingerprints can be used to search for the unknown molecule in a chemical structure database. In the training phase, each spectrum of the training set is transformed into a feature vector. For each structural property of the fingerprint, feature vectors are marked as possessing it or not. A support vector machine (SVM) ML technique is trained to predict which structural features of the fingerprint are present in a compound from its spectra. In a related work, Shen et al. [83] reported a kernel-based ML method to predict molecular fingerprints from MS data and fragmentation trees [84]. Fragmentation trees can be considered as an annotated representation of the original fragmentation mass spectrum. Experiments on two large reference datasets, METLIN and MassBank, have shown that the inclusion of fragmentation tree kernels significantly increases the molecular fingerprint prediction accuracy [83]. A further improvement was achieved by combining more kernels, more fingerprints, and a refined fingerprint similarity scoring (CSI:FingerID) [85].

#### 3.1.2. Genome-Guided

The fast development of genome sequencing methods and the exponentially rising number of genome sequences available revolutionized almost every aspect of biology, including NP research. In spite of the large diversity of secondary metabolites, the structures of the involved enzymes are much conserved, making it possible to mine genomes for genes encoding biosynthetic enzymes [86]. The key feature of the renaissance of NP drug discovery would be to turn the ad-hoc process of discovering NPs into a high-throughput pipeline yielding many thousands of new small molecules from microbes [87]. However, more than 10 years after the first Streptomyces genomes were sequenced [88,89], this promise has not yet been realized. Indeed, over the last decade not more than a few hundred molecules have been discovered using genome mining, and many of those molecules were so challenging to discover that the process would be difficult to generalize and automate.

**Figure 6:**
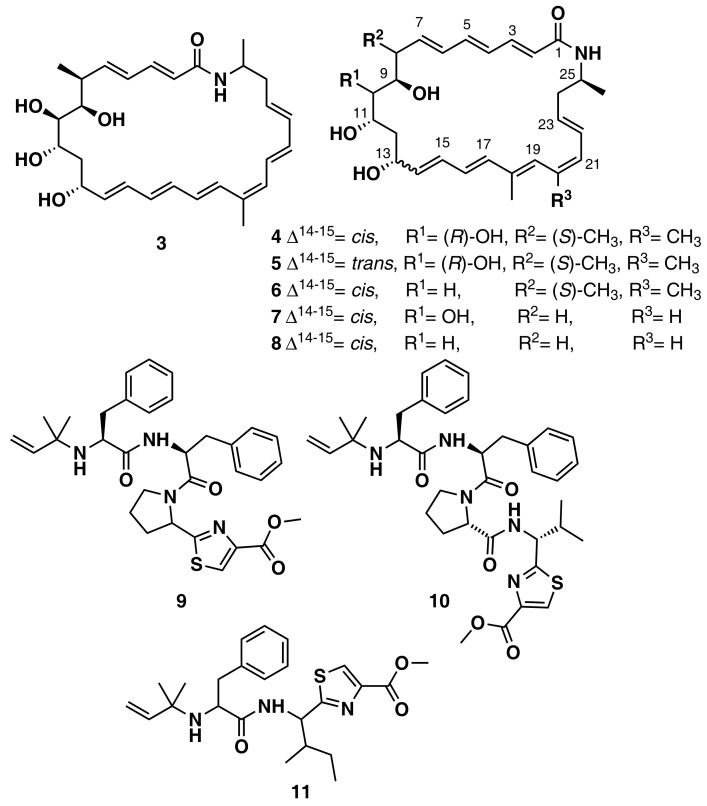
Chemical structures of MNPs identified using genome mining approaches.

Ziemert et al. reviewed the evolution of genome mining in microbes and included an extensive list of examples where genome mining has directly led to the identification of metabolites [86]. For example, the discovery of the polyene macrolactam salinilactam A (**3**) (Figure 6) demonstrates the powerful interplay between genomic analysis and traditional studies of NP chemistry. The salinilactam gene cluster is the biggest gene cluster detected by bioinformatic analysis in the marine actinomycete Salinispora tropica CNB-440 genome [90]. The detection of the compound was possible based on putative structural features (characteristic UV chromophores) suggested by the initial inspection of the partial gene cluster. Then, the structural fragments and the molecular formula obtained by MS for an isolated product suggested a 10-module polyketide synthase (PKS) enzyme responsible for the biosynthesis of the compound, which facilitated assembly and therefore closure of the genome. Finally, further bioinformatic analysis of enzymatic domains refined the structure elucidation of the compound. A similar strategy was followed by Schulze et al. [91] and enabled the discovery of a family of macrolactams from a marine actinomycete, Micromonospora sp. (lobosamide A (**4**), B (**5**), and C (**6**) are illustrated in Figure 6), as well as mirilactam A (**7**) and B (**8**) from a distantly related actinobacterium, Actinosynnema mirum. A genome mining study reported the identification of 31 cyanobactin gene clusters from 126 genomes of the marine cyanobacteria Microcystis aeruginosa PCC 9432 and Oscillatoria nigro-viridis PCC 7112 [92]. Cyanobactins are a growing family of cyclic ribosomal peptides produced by cyanobacteria, which have exhibited cytotoxic activity against cancer cell lines as well as antiviral, antimalarial, and allelopathic activities. Bioinformatic analysis of the genomes predicted that the strains produce cyanobactins with chain lengths of 3, 4, and 5 amino acids and containing thiazoles (the core encoded a cysteine and the gene cluster encoded heterocyclase and oxidase enzymes). Extensive chemical analyses demonstrated that some cyanobacteria produce short linear peptides with a chain length ranging from three to five amino acids. Three novel linear peptides, aeruginosamide B (**9**) and C (**10**) and viridisamide A (**11**) (Figure 6), were isolated, which were N-prenylated and O-methylated on the N and C termini, respectively. 

**Figure 7:**
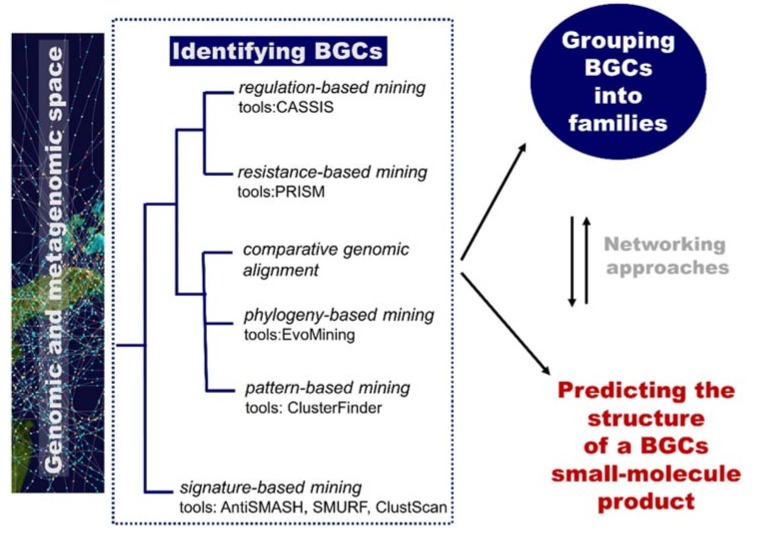
The role of computational methodologies in genome mining for natural product discovery. BGC, biosynthetic gene cluster.

Of particular relevance for the computational identification of genes encoding metabolic pathways is the fact that they are typically chromosomally adjacent, forming biosynthetic gene clusters (BGCs). These BGCs encode all the biosynthetic machinery to produce, process, and export a specialized metabolite (enzymes, regulatory proteins, and transporters) [87]. They are useful targets for mining genomes (to discover new metabolites) based on knowledge of homologous genes and rules/patterns extracted from them. Plenty of computational tools are available for researchers to mine genetic data and to connect them to known secondary metabolites. An overview of computational tools for genome mining is displayed in Figure 7. Reviews include references [86] and [87]. The Secondary Metabolite Bioinformatics Portal (SMBP) website at http://www.secondarymetabolites.org maintains a catalogue of available software, databases, and hand-curated links to major resources used in the field [93]. We are currently far from the initial simple comparison techniques using manually constructed lists of genes as query sequences, such as the sequence-based comparison with BLAST [94] or profile-based tools, such as HMMer [95]. Nowadays, comprehensive software resources are available and typically classified into two categories: low-novelty methods using profiles of known and highly conserved biosynthetic machineries (e.g., polyketide synthases or non-ribosomal peptide synthetases domains) and high-novelty methods detecting new classes of gene clusters (Figure 7). Examples of software implementing low-novelty methods are ClustScan [96], SMURF [97], and antiSMASH [98]. The most comprehensive tool, antiSMASH, can detect more than 20 classes of pathways. High-novelty methods include pattern-based mining, phylogeny-based mining, comparative genomic alignment, resistance-based mining, and regulation-based mining.

The ClusterFinder software implements a pattern-based mining strategy (based on a hidden Markov model-based probabilistic algorithm) and aims to identify gene clusters of both known and unknown classes [99]. Instead of looking for specific individual signature genes, ClusterFinder recognizes patterns of broad gene functions encoded in a genomic region. In a study of secondary metabolites of proteobacteria, ClusterFinder enabled the identification of a large, previously unrecognized family of gene clusters that encode the biosynthesis of aryl polyenes [99].

Phylogeny-based mining incorporates evolutionary principles into gene mining: enzymes evolve in their substrate specificity and acquire new metabolic functions keeping detectable relationships with ancestral primary metabolic enzymes [100]. Cruz-Morales et al. [100] reported the use of EvoMining, a phylogeny-based mining approach, to discover a biosynthetic pathway for arseno-organic metabolites in Streptomyces coelicolor and Streptomyces lividans. The EvoMining method was implemented in a standalone tool distributed as a docker image developed by the EvoDivMet lab and has been made available at https://github.com/nselem/EvoMining.

Takeda et al. reported a comparative genomic alignment methodology based on the assumption that secondary metabolism genes are highly enriched in nonsyntenic blocks; a biosynthetic gene cluster can be detected by searching for a similar order of genes and their presence in nonsyntenic blocks. This approach enabled the detection of biosynthetic gene clusters without core genes, e.g., the kojic acid biosynthesis gene cluster of Aspergillus oryzae [101].

PRISM (PRediction Informatics for Secondary Metabolomes) is an open-source web application for the genomic prediction and dereplication of nonribosomal peptide and type I and II polyketide chemical structures [102]. This software is based on hidden Markov models that can predict not only genes involved in NP biosynthesis but also in antibiotic resistance. Genes encoding resistance functions can lead to the identification of enzymes for the biosynthesis of new antibiotics [86] as bacteria producing antibiotics may have their own resistance mechanisms to avoid self-destruction. Such a resistance-based approach is illustrated with the work of Moore and co-workers [103] that screened the genomes of 86 marine Salinispora bacterial genomes and prioritized an orphan polyketide synthase–nonribosomal peptide synthetase hybrid BGC (tlm) with a putative fatty acid synthase resistance gene. The expression of the tlm and the related ttm BGCs in Streptomyces hosts led to the production of unusual thiotetronic acid antibiotics.

Finally, CASSIS is an example of a regulation-based mining tool that exploits the idea of co-regulation of the cluster genes and assumes the existence of common regulatory patterns in the cluster promoters; the method searches for “islands” of enriched cluster-specific motifs in the vicinity of anchor genes [104]. This strategy can be particularly useful in fungi as genes of the same BGC are highly co-regulated [86].

### 3.2. Computer-Assisted Structure Elucidation (CASE)

Fully automated structure elucidation from spectroscopy data has been achieved for small organic molecules, from 1D NMR data, or for complex NPs using 2D NMR data. CASE expert systems have been developed for over 40 years. Currently available packages include the open source Seneca platform [105,106], the commercial ACD/Structure Elucidator Suite [107,108,109], LSD [110], and CMC-se (http://www.bruker.com). Here, we review some recent achievements of CASE expert systems.

Troche-Pesqueira et al. reported enhanced CASE procedures for the determination of the relative configuration of NPs, which starts from the molecular formula and combines conventional one-dimensional (1D) and 2D NMR spectra with residual dipolar couplings (RDCs) and/or residual chemical shift anisotropy (RCSA) [111,112]. The employment of RDC data in conjunction with a CASE program automated the determination of relative configurations in molecules of medium complexity and a moderate degree of flexibility, such as naltrexone, 10-epi-8-deoxycumambrin, strychnine, eburnamorine, yohimbine, and N-methylcodeine. The pool of diastereoisomeric candidates was enumerated and the conformational space was explored for flexible molecules in the process of identifying the structure that best agrees with the RDC data. Moreover, the authors demonstrated that the assignment of absolute configurations can also be incorporated by comparison of experimental and density functional theory (DFT)-calculated vibrational or electronic circular dichroism (VCD or ECD) curves [111].

**Figure 8:**
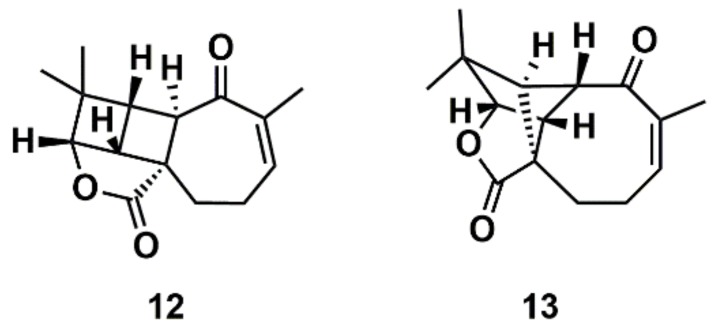
Proposed structure of aquatolide (**12**) and the corresponding revised structure (**13**).

Liu et al. [112] proposed a protocol comprising the confluence of capabilities embodied by CASE methods, DFT calculations, and measurement of anisotropic NMR parameters (RDCs and RCSA) aiming at the growing general problem of structural mischaracterization. The authors demonstrated that the combination of RDCs and RCSAs provides a powerful orthogonal mean of confirming not only the relative configuration of a given stereocenter, but also the overall molecular structure and atomic connectivity of a molecule [112]. The protocol was applied to several examples of revised structures, including aquatolide, a sesquiterpene lactone isolated from the hexane extract of Asteriscus aquaticus. In 1989, a very rare ladderane moiety was proposed [113] for the aquatolide (12) (Figure 8). However, more recently, the proposed chemical structure of the aquatolide (**12**) was revised on the basis of quantum-chemical calculations and NMR experiments to the unusual core structure (**13**) (Figure 8) [114]. The revised structure of aquatolide was subsequently confirmed by X-ray crystallography [114] and by total synthesis [115]. Liu et al. compared the experimental and back-calculated RDC/RCSA data for the model structures (**12**) and (**13**) (Figure 8) and readily established that the revised structure (**13**) is in best agreement with the data [112].

**Figure 9:**
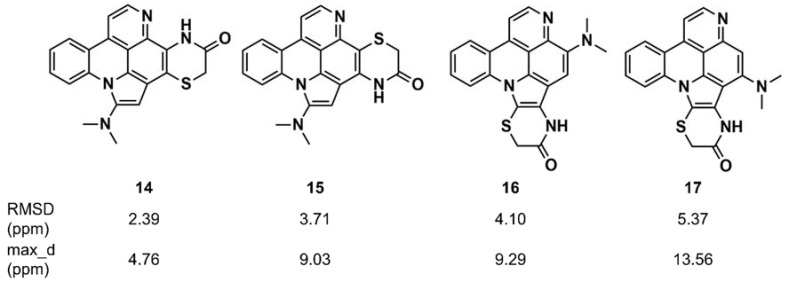
Root mean square deviation (RMSD) and maximum chemical shift deviation between experimental and density functional theory (DFT)-calculated carbon chemical shifts [118] for four isomers of cycloshermilamine D suggested by Computer-Assisted Structure Elucidation (CASE) analysis.

Synergistic combinations of CASE algorithms and DFT calculations of chemical shifts have been reported that broaden the range of amenable structural problems to encompass proton-deficient molecules, molecules with heavy elements (e.g., halogens), conformationally flexible molecules, and configurational isomers [116,117,118]. Buevich and Elyashberg [118] illustrated this approach with previously established structures; one example is cycloshermilamine D (**14**) (Figure 9), a pyridoacridine alkaloid isolated from the marine tunicate Cystodytes violatinctus [119]. The ACD/Structure Elucidator system processed the experimental data, consisting of the molecular formula, 1D proton and carbon spectra, and 2D NMR data (COSY, HSQC, and HMBC), and yielded 263 candidate structures. The four top candidates included the structure of cycloshermilamine D at the first position, but the other three candidates had very similar sets of carbon chemical shift deviations. DFT calculations of carbon chemical shifts for the four structures were performed at the mPW1PW91/6-311 + G(2d,p) level of the theory, unequivocally showing that the first structure had the lowest root mean square deviation (RMSD) (^13^C) and the smallest maximum chemical shift deviation, which convincingly supported the structure of cycloshermilamine D without any additional experimental data.

## 4. Computer-Aided Drug Design (CADD)

Computer prediction of biological activities of MNPs is required to guide decisions concerning the in vivo and in vitro testing of isolated NPs and extracts, to assist in the design of bioactive NP derivatives, and to virtually screen databases of known or proposed NPs. Additionally, the regions of the chemical space encompassing NPs are recognized as promising for the invention of new drug leads as they result from the evolution of chemical structures during millions of years for optimum performance of biochemical machineries [120]. Furthermore, advances have been reported on computational methodologies to explore global networks connecting active compounds and their targets [121,122,123,124], to simulate interactions between ligands and binding sites [125,126,127,128,129,130,131,132], and to establish structure-activity relationships with NPs and MNPs [133,134,135,136,137]. Available ADMET predictors for several endpoints, e.g., human intestinal absorption, Caco2 (heterogeneous human epithelial colorectal adenocarcinoma), cell permeability, or blood brain barrier permeability, are often applied in screening procedures to filter out molecules with undesirable properties [132,134,138].

### 4.1. Ligand-Based (LB)

Ligand-based methodologies are useful to discover new lead compounds when sets of active molecules are known for specific targets. Developed strategies include similarity searches in databases of molecules, structure alignment for the identification of pharmacophores and virtual screening, and ML algorithms to establish Quantitative Structure-Activity Relationships (QSARs), predict properties of candidates, and guide the design of new molecules.

Dineshkumar et al. [139] performed target prediction for sporolides A and B using LB pharmacophore screening against known inhibitors and drugs. These NPs are polycyclic macrolides from the obligate marine actinomycete Salinispora tropica. Eight pharmacophore features were identified in sporolides A and B: six H-bond acceptors, one hydrophobic group, and one aromatic ring [139]. The three-dimensional (3D) models were generated and the pharmacophore pattern was used to screen the public Binding Database with 400,000 known ligands. A small group of targets was retrieved bearing similar pharmacophore features, and these were further explored with structure-based methods. HIV-1 reverse transcriptase chain A emerged as a predicted target. In vitro testing showed that sporolide B significantly reduced the activity of HIV-1 RT and could be a possible drug candidate for HIV and other retroviral viruses [139]. The same lab later reported a similar computational study for the MNPs salinosporamides A, B, and C from the same source and concluded that the glucocorticoid receptor and methionine aminopeptidase 2 could be new drug targets, suggesting possible antiinflammatory and anticancer activities of salinosporamides [140].

Waldmann and co-workers [141] suggested, from a statistical analysis of the structural classification of NPs, that more than half of all NPs have just the right size (i.e., a van der Waals volume between 300 and 800 Å^3^) to serve as a starting point for hit and lead discovery. Indeed, Pereira et al. [142] have also observed, in a subset of PubChem and AntiMarin, a correlation between active compounds and three- or four-ring compounds with a van der Waals volume between 300 and 800 Å^3^. Ertl et al. [120] developed a NP-likeness score to measure the similarity between a molecule and the structural space covered by NPs. A NP-likeness score was incorporated in SENECA, an open-source CASE platform, significantly improving the ranking of candidates in structure elucidation of metabolites [106]. Similar approaches can be used in virtual screening, in prioritization of compound libraries toward NP-likeness, and in the design of building blocks for the synthesis of NP-like libraries [120]. More recently, Shang et al. [143] analysed the differences between terrestrial and marine NPs using chemoinformatics methods on a data set with 32,937 MNPs and 132,071 terrestrial NPs. The authors observed a trend for MNPs to have lower solubility, longer chains and larger rings, more halogens (especially bromine), and nitrogen. MNP scaffolds are less represented in databases of known ligands, which agrees with the fact that MNPs have been less exploited in drug discovery projects and suggests their greater potential in developing new drugs.

**Figure 10:**
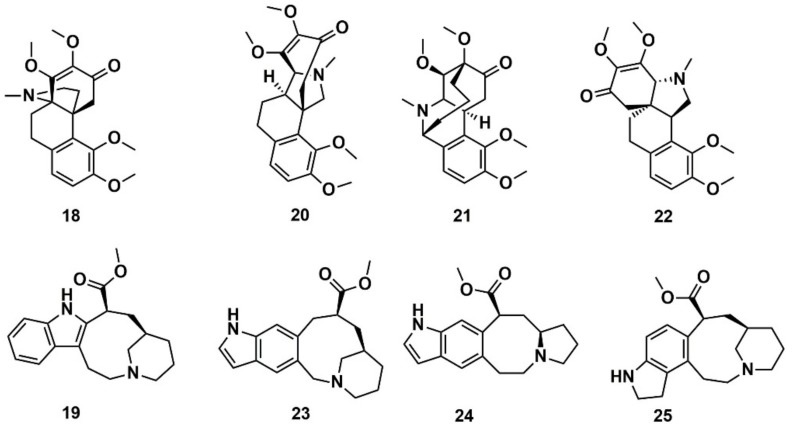
Chemical structure of hasubanonine (**18**) and vincadine (**19**) as well as their designed analogs, **20**–**22** and **23**–**25**, respectively.

Reymond and co-workers [144] enumerated possible organic saturated or aromatic ring systems with up to 4 cycles and 14 atoms to obtain the so-called GDB4c database containing 916,130 ring systems. This was further processed to generate all possible stereoisomers, yielding a GDB4c3D database with 6,555,929 compounds. Almost all of these ring systems are unknown and represent chiral 3D macrocycle structures; included are many polycyclic scaffolds reminiscent of NPs. The database is a useful resource for similarity and pharmacophore searching on the basis of known NPs. It is available for download at www.gdb.unibe.ch together with interactive tools for data mining. The authors illustrated the platform by searching for similar structures of the NPs hasubanonine (**18**) and vincadine (**19**) (Figure 10). The results enabled the identification of similar 3D structures with new ring systems and led to the proposal of the six new analogs **20**–**22** and **23**–**25**.

NPs often contain macrocycles, which are problematic structures for CADD due to their size (generally >500 MW) and conformational complexity. Low-energy conformations must be identified to model conformation-dependent properties. Macrocyclic polyketides are medically and biologically important NPs characterized by structural and functional diversity [145]. Wang et al. proposed an improved dihedral angle-based macrocycle conformational sampling method and evaluated its performance with a data set of 37 polyketides with 9−22 rotatable bonds in the macrocyclic ring for which crystal structures were available [145]. The protocol was able to reproduce the crystal structure of polyketides’ aglycone backbone within an RMSD of 0.50 Å for 31 out of 37 polyketides [145].

**Figure 11:**
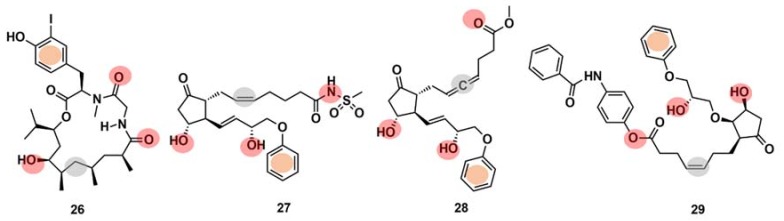
Chemical structures of doliculide (**26**) and three known prostanoid receptor ligands, sulprostone (**27**), enprostil (**28**), and GR63,799 (**29**). The pharmacophore features are indicated in the chemical structures by colored dots: red, hydrogen-bond donors; grey, lipophilic interaction centers; and orange, aromatic centers.

Drug interaction with multiple targets is a cause of drug side effects [146], but it can also be used to increase drug efficacy [147], repurposing [121,148], and design multitarget molecules [149]. Systematic experimental identification of drug targets for NPs or known drugs at the human proteome level is not feasible for the thousands of compounds currently available. Therefore, the development of computational tools to predict the targets of new or known molecules in a systematic way is of high interest [121]. It has been claimed [150] that ML models can point to potential target families and sometimes even to the target subtypes of approximately one-third of the NPs identified to date. Schneider et al. [150] computationally identified and biochemically confirmed an unknown, high-affinity macromolecular target of doliculide (**26**) (Figure 11), an MNP that is produced by the sea hare Dolabella auricularia. The authors performed automated target prediction with the SPiDER protocol for both doliculide, an NP with strong actin-polymerizing and anticancer activities, and 134 intermediates and precursors of a total synthesis. The SPiDER protocol performs a projection of query compounds, represented by pharmocophore topological descriptors, onto a self-organizing map (SOM) consisting of 120 receptive fields, which was previously trained with pharmacologically active reference compounds and their known targets [149]. The prostaglandin receptors (e.g., EP2, EFP3, and EP4) were predicted as targets not only for doliculide itself but also for most of the synthesis intermediates (100 out of the 134). Doliculide represented a novel chemotype among G-protein-coupled receptor ligands. A flexible three-dimensional pharmacophore alignment was also performed between doliculide (**26**) and three well-studied, non-selective prostanoid agonists (**27**–**29**) (Figure 11). The alignment revealed that the four compounds contain a total of five common pharmacophore points.

Network-based approaches have also been used for the systematic identification of drug−target interactions (DTIs) and assessment of drug safety profiles [121]. Fang et al. [121] proposed a statistical network model to predict new drug targets and anticancer indications of NPs. A global drug−target network was reconstructed that linked molecules, substructures, and targets and resulted in 7314 interactions connecting 751 targets and 2388 NPs. New interactions are predicted from the substructures of query compounds. The authors computationally identified multiple anticancer indications for several typical NPs with a new mechanism of action (MOA) across 13 cancer types. For example, naringenin (a flavanone mainly found in grapefruit, oranges, and tomatoes), disulfiram (an FDA-approved carbamate derivative for the treatment of chronic alcoholism), and metformin (a biguanide oral agent for treating type 2 diabetes) showed six (bladder, lung, uterine, colon, prostate, and breast), five (breast, colon, lung, thyroid, and uterine), and two (breast and ovarian) new MOAs, respectively [121].

Linear regressions and ML algorithms are well-known to establish QSARs, which are trained with available experimental data and molecular descriptors encoding structural features to make predictions for new molecules. Here, we describe recent examples of QSAR models used to estimate biological activities and ADMET properties of MNP. Davis and Vasanthi [134] retrieved 157 compounds from the Seaweed Metabolite Database of marine algal secondary metabolites (http://www.swmd.co.in) and developed a QSAR approach concerning anticancer activity against six different cancer cell lines: MCF-7 (human breast adenocarcinoma), A431 (human epithelial carcinoma), HeLa (human cervical adenocarcinoma), HT-29 (human colon adenocarcinoma grade II), P388 (murine leukemia), and A549 (human lung epithelial adenocarcinoma). The QSAR process was used to identify relevant structural features and to support the choice of protein kinase B (PKB) targets for further structure-based studies. ADMET predictions were later used to select a lead compound. A QSAR approach was also pursued by Knight et al. [135] using 43 synthetic derivatives of the marine alkaloid tambjamine to model transmembrane anion transport activity. The data set comprised bipyrrole core derivatives with three substitution patterns. A parabolic dependence of the anionophoric activity was observed with lipophilicity, which was quantified in two-, three-, and four-parameter linear model equations.

The quest for new antimalarial drugs has also led to the investigation of MNPs with QSAR methods [136,137]. Aswathy et al. [136] analyzed 42 analogs of the natural product thiaplakortone-A, which was found in the Australian marine sponge Plakortis lita and is active against chloroquine-sensitive and chloroquine-resistant Plasmodium falciparum. Several QSAR models, including both 2D and 3D QSAR, were developed, and the results were combined with simulated interactions with the P. falciparum calcium-dependent protein kinase 1 protein to design and screen new virtual molecules. Three new molecules were proposed as leads to potential anti-malarial drugs. In a different approach, quantitative relationships were established between thermodynamics/electronic properties calculated by DFT methods and antimalarial activity [137]. Linear regressions were performed with a data set of 14 sponge metabolites–bromopyrrole alkaloids. The best model (r^2^ = 0.97, Q^2^ = 0.86, F = 41.85) was obtained using the molecular descriptors entropy, dipole moment, molecular polarizability, energy of the highest occupied molecular orbital (HOMO), softness, and electrophilicity index [137]. The HOMO also performed remarkably well in discriminating overall biological activity of MNP and microbial NPs [151].

### 4.2. Structure-Based (SB)

Molecular docking has been the major SB methodology to predict affinities to macromolecular targets, to interpret binding modes, and to assist in the design of drug leads. Several recent publications illustrate the application of the method to MNPs [127,128,129,138,152,153,154,155,156,157,158,159,160,161,162,163,164,165,166,167,168,169,170,171], and some representative examples are here described.

**Figure 12:**
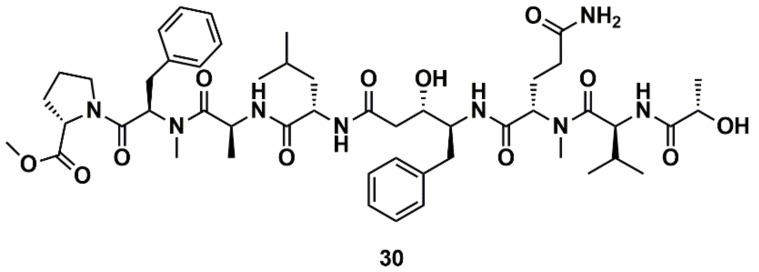
Chemical structures of tasiamide B (**30**).

Liu et al. [129] designed, synthesized, and evaluated 19 new derivatives of the MNP tasiamide B (**30**) (Figure 12) as inhibitors of BACE1, a potential therapeutic target for Alzheimer’s disease. Tasiamide B is an acyclic peptide containing a statine-like unit and several aminoacid residues. The exploration of structure–activity relationship (SAR) with truncated derivatives identified a core structure as well as a free carboxylic acid group important for inhibitory activity. The conclusions were supported by a docking simulation.

**Figure 13:**
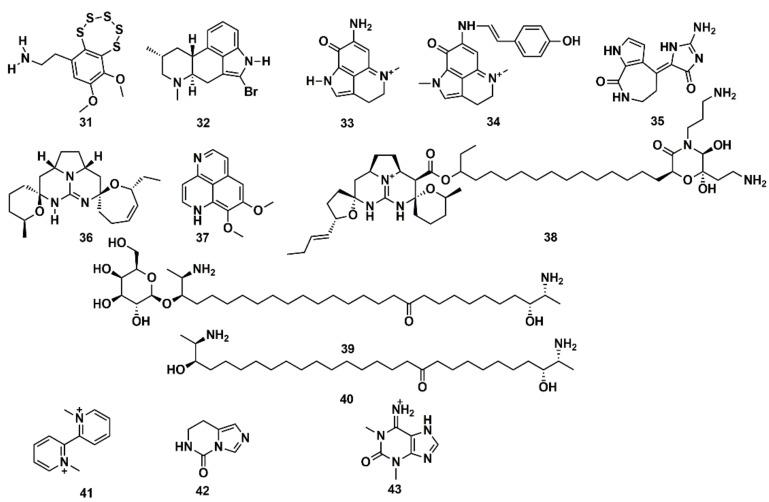
Chemical structures of MNPs predicted to have high (**31**–**38**) and low (**39**–**43**) affinity to *Lymnaea stagnalis* AChBP using three docking approaches.

SB computational studies and in vitro experimentation were combined to elucidate the molecular target of 13 low molecular weight MNPs from marine sponges and ascidians. Some are bioactive and the structural similarity to diverse cholinergic ligands anticipated their possible activity towards nicotinic acetylcholine receptors (nAChRs) [127]. In silico docking to the Lymnaea stagnalis acetylcholine-binding protein (AChBP), a model for the ligand-binding domains of nAChRs, was carried out. High affinity was predicted for some compounds, such as the polysulfide varacin (**31**) and the seven alkaloids pibocin (**32**), makaluvamines C and G (**33**, **34**), debromohymenialdesine (**35**), crambescidin 359 (**36**), aaptamine (**37**), and monanchocidin (**38**), while low efficiency of interaction was suggested for other compounds, such as the two sphingolipids rhizochalin (**39**) and its aglycone (**40**) as well as the three alkaloids 1,1′-dimethyl-[2,2′]-bipyridyldiium salt (**41**), 7,8-dihydroimidazo-[1,5-*c*]-pyrimidin-5(6*H*)-one (**42**), and 1,3-dimethylisoguaniniium hydrochloride (**43**) (Figure 13). The conclusions from computer modelling were verified by radioligand analysis. Nicotinic acetylcholine receptors exhibit multiple conformational states: resting (channel closed), active (channel open), and desensitized (channel closed). Homology modelling was used by Mallipeddi et al. [172] to generate structures of the Torpedo californica α_2_βδγ nAChR that initially represent the resting state and the desensitized state. Molecular dynamics (MD) simulations were performed on the extracellular ligand binding domain on each nAChR conformational state with and without the agonist anabaseine present in each binding site. Anabaseine (a bipyridine derivative) is a marine alkaloid toxin that acts as an agonist on most nAChRs in the central nervous system. The MD simulations revealed that in the presence of agonist, loop C was drawn inward and attained a more stable conformation [172].

Protein kinases and acetylcholinesterase (AChE) are potential targets for the treatment of Alzheimer’s disease (AD). Llorach-Pares et al. reported a molecular docking investigation of meridianins A–G (a group of indole alkaloids isolated from the marine tunicate Aplidium) towards protein kinases in order to assist in the future development of anti-AD drugs [138]. Post-processing of docking results was performed with MD simulations. The results provided information concerning binding mode, strength, and selectivity and were complemented with ML predictions of ADMET properties. Botic et al. described four brominated pyrroloiminoquinone alkaloids (discorhabins) isolated from Latrunculia sp. sponges collected near the Antarctic Peninsula and their promising activity as reversible competitive inhibitors of cholinesterases. Docking calculations with different AChEs revealed the involved interactions in the active sites and provided further support for the experimental data [152].

Wang et al. [165] studied the antibacterial activity of a novel anthraquinone, 2-(dimethoxymethyl)-1-hydroxyanthracene-9,10-dione, together with nine known anthraquinone derivatives isolated from the marine-derived fungus Aspergillus versicolor. The novel molecule showed strong inhibitory activities against MRSA ATCC 43300, and MRSA CGMCC 1.12409 (with MIC values of 3.9 and 7.8 μg/mL, respectively). Molecular docking studies predicted that the new anthraquinone binds to the AmpC β-lactamase and topoisomerase IV enzymes, which could explain its antimicrobial properties. It bound to DNA topoisomerase IV receptor similarly to a co-crystallized ligand and with lower binding energy. The same was observed in the β-lactamase binding site.

Chen et al. [126] reported the synthesis of a series of novel 1,2-dithiolan-4-yl benzoate derivatives inspired by bruguiesulfurol, a marine cyclic disulphide, and their in vitro inhibitory activity against the enzyme protein tyrosine phosphatase 1B (PTP1B), a validated target for the treatment of diabetes and obesity. An SAR analysis assisted by molecular docking allowed the authors to reveal the derivative with a 2,5-dibromidebenzyloxy terminal moiety as the most potent PTP1B inhibitor among all 11 derivatives (IC_50_ = 0.59 μM), with improved activity compared to the original hit [126]. Inhibitors of the same enzyme were isolated from the marine brown alga Sargassum serratifolium. Three plastoquinones (sargahydroquinoic acid, sargachromenol, and sargaquinoic acid) exhibited dose-dependent inhibitory activity against PTP1B (IC_50_ range of 5.14–14.15 µM). In addition, sargachromenol and sargaquinoic acid also showed dose-dependent inhibitory activity against α-glucosidase (IC_50_ 42.41 and 96.17 µM, respectively). The results of docking simulations indicated a high affinity and tight binding capacity towards the active site of the PTP1B and α-glucosidase enzymes [157]. Docking was also used by Xu et al. [158] to understand the high activity against PTP1B (IC_50_ 0.84 µM) of a marine-derived bromophenol compound isolated from the red alga Rhodomela confervoides.

Twelve pyrrole alkaloid derivatives, isolated from an Australian marine sponge, Ianthella sp., were evaluated as inhibitors of ATP binding cassette (ABC) transporters, a potentially useful activity to overcome multi-drug resistance of cancer cells [128]. One of them, lamellarin O, was found to be a potent selective inhibitor of the BCRP ABC transporter. An SAR analysis covering the 12 MNPs and 6 synthetic analogues was supported by in silico docking studies and identified structural elements of the inhibitory pharmacophore, including a methoxy-acetophenone, a carboxylic ester, and two phenolic residues.

Cen-Pacheco et al. [153] applied molecular docking to understand the different activity of two novel squalene derivatives, isolated from the red seaweed Laurencia viridis, as inhibitors of Ser-Thr protein phosphatase type 2A (PP2A). This enzyme has several functions in cells and is a tumour promoter and suppressor, making it a potential target for new anticancer drugs. The two novel squalene derivatives, (+)-longilene peroxide and (+)-prelongilene, were evaluated for their ability to inhibit PP2A. While (+)-longilene peroxide is an inhibitor (IC_50_ 11.3 μM ±1.4), (+)-prelongilene is inactive at a concentration of 100 μM. Docking simulations onto the PP2A enzyme-binding region revealed that, although the two compounds have similar binding modes, the first establishes several favourable contacts that are not observed with the second, and the second has unfavourable contacts with several residues. The results indicated that the additional allylic hydroperoxide group at C-2 in (+)-longilene peroxide is responsible for key hydrogen bonds and appears to be the factor leading to the differences in bioactivity [153]. Similarly, Cruz et al. rationalized the different activity against protein phosphatase 1 and 2A of two new marine brominated bis(indole) alkaloids, dragmacidins I and J, with docking into the binding pocket of PP1 [154]. Structure-based virtual screening enabled Xin et al. to discover new DNA topoisomerase I (Topo I) inhibitors, which are potential antitumor agents. A collection of 138 structures from low-cytotoxic or non-cytotoxic coral-derived fungi and plants were docked to the central catalytic domain of the Topo I–DNA complex and the 27 molecules with the most favourable predicted interactions were evaluated in vitro. Among these, four compounds showed activity at 25 μM and two compounds were active at 5 μM [155].

The ability of reverse docking for target fishing of MNPs was evaluated by Chen et al. using 40 marine compounds with known antitumor activities and known target proteins but without their crystal structure determined [159]. A database of anti-tumor proteins was constructed with 470 crystal structures corresponding to 150 different target proteins. After docking the 40 MNPs to the proteins in the database, it was observed that, although the predicted binding energy for a given ligand to its known target is usually not the lowest, 55% of the compounds have their reported target ranked in the top 20, and 30% in the top 10. It is noted that the compounds may have multiple targets and some of them may have not been discovered and reported yet [159].

In general, the LB and SB methods are complementary and were used as such in several of the works here cited [134,136,139,140]. In a comparative study of docking and similarity searches (based on 2D and 3D fingerprints), Avram et al. concluded that fusing the results obtained by the two approaches can enhance the probability to find new chemotypes in virtual screening [173]. Ebrahim and Sayed [131] reported the exploration of a MNP-based mini-library comprising 71 molecules with diverse scaffolds (e.g., macrolides, sesquiterpenes, diterpenes, sesterterpenes, triterpenes, and alkaloids). They were submitted to the Lilly’s Open Innovation for Phenotypic Drug Discovery (PD2-OIDD) program for biological screening after successfully passing the initial online bioinformatics screen (https://openinnovation.lilly.com/dd/). The bioinformatics filter calculates molecular descriptors and evaluates drug-like characteristics. Among the surviving 38 MNPs and semisynthetic derivatives, several compounds showed promising results in primary and secondary angiogenesis screening modules and minimal cytotoxicity at relevant doses. According to the authors, molecular modelling and docking experiments aided in understanding molecular binding interactions, identifying pharmacophoric epitopes, and deriving structure-activity relationships of active hits.

Finally, Skariyachan et al. applied a computational workflow to identify possible lead molecules against the Ebola virus among compounds from microbial symbionts associated with marine sponges [132]. The procedure included the calculation of drug likeness and ADMET properties followed by docking of the selected molecules against the VP40 target of Ebola virus. Lead molecules, such as gymnastatin G (a sterol derivative with anti-leukemia activity), sorbicillactone A (an alkaloid derivative with anti-leukemia and anti-HVI-1 activities), marizomib (a β-lactone-γ-lactam derivative with anti-proteasome activity), and daryamide C (a polyketide derivative with anticancer activity against the human colon carcinoma cell line), were proposed as possible inhibitors against the VP40 matrix protein of the Ebola virus [132].

## Figures and Tables

**Table 1:** Essential features of selected databases for NPs dereplication and CADD.

Database	Compounds ^6^		Taxo. ^7^	Bioact. ^8^	Targets ^9^	Spec. Data ^10^
	Total	NPs				
CAS/SciFinder ^1^	9.0 × 10^7^	>283,000	+	+	-	-
ChemSpider ^2^	5.9 × 10^7^	>13,800	-	+	-	-
PubChem ^2^	9.3 × 10^7^	4.4 × 10^5^	-	+	+	+ ^10^
ChEMBL ^2^	1.7 × 10^6^	>75,000	-	+	+	-
REAXYS ^1,2^	1.1 × 10^8^	>215,000	+	+	-	-
ZINC ^2,5^	1.2 × 10^8^	>44,000	-	+	+	-
LOPAC ^3,5^	1280	?	-	+	+	-
Prestwick ^3,5^	1280	?	-	+	+	-
ACD/NMR DB ^4^	>322,000	>50,000	-	-	-	+ ^10.2^
NMRShiftDB ^4^	43,440	?	-	-	-	+ ^10.2^
Massbank ^4^	>15,000	>2500	-	-	-	+ ^10.3^
ReSpect ^4^	-	>3595	-	-	-	+ ^10.3^
METLIN ^4^	-	75,000	-	-	-	+ ^10.3^
GNPS ^4^	22,644	>3000	+	-	-	+ ^10.3^
NaprAlert ^4^	-	>155,000 ^12^	+	+	-	+ ^10.1^
DNP ^4^	-	>270,000	+	+	-	+ ^10.1^
DMNP ^4^	-	>30,000	+	+	-	+ ^10.1^
MarinLit ^4^	-	>29,000	+	+	-	+ ^10^
AntiBase ^4^	-	43,743	+	+	-	+ ^10^
StreptomeDB ^4^	-	3991	+	+	-	+ ^11^
NPCARE ^2,4^	-	6578 ^12^	+	+	+	-

^1^ Comprehensive compilation of information on NPs with no specific application in view; ^2^ Particularly suitable for CADD applications; ^3^ Chemogenomic libraries that were conceived for cell-based high-throughput screening (HTS) assays, but are also suitable for CADD applications; ^4^ Suitable for dereplication applications; ^5^ Commercially available compounds; ^6^ When possible an estimate number of NPs in the database is given; ^7^ Taxonomy; ^8^ Bioactivity; ^9^ Biological targets; ^10^ Spectral data comprising ^10.1^ UV, ^10.2^ NMR, and ^10.3^ MS data; ^11^ Predicted ^1^H/^13^C NMR and MS spectra; ^12^ Comprising extracts, NPCARE contains 2566 fractional extracts isolated from 1952 distinct biological species, including plants, marine organisms, fungi, and bacteria.
